# The crude oil biodegradation activity of *Candida* strains isolated from oil-reservoirs soils in Saudi Arabia

**DOI:** 10.1038/s41598-022-14836-0

**Published:** 2022-06-23

**Authors:** Fatimah Al-Otibi, Rasha M. Al-Zahrani, Najat Marraiki

**Affiliations:** Department of Botany and Microbiology, College of Science, King Saud University, P.O. Box 22452, Riyadh, 11495 Saudi Arabia

**Keywords:** Ecology, Microbiology, Biogeochemistry, Ecology, Environmental sciences, Natural hazards

## Abstract

Crude oil (petroleum) is a naturally occurring complex composed of hydrocarbon deposits and other organic materials. Bioremediation of crude oil-polluted sites is restricted by the biodiversity of indigenous microflora. They possess complementary substrates required for degrading the different hydrocarbons. In the current study, four yeast strains were isolated from different oil reservoirs in Riyadh, Saudi Arabia. The oil-biodegradation ability of these isolates showed variable oxidation effects on multiple hydrocarbons. The scanning electron microscopy (SEM) images showed morphological changes in *Candida* isolates compared to the original structures. The drop-collapse and oil emulsification assays showed that yeast strains affected the physical properties of tested hydrocarbons. The content of biosurfactants produced by isolated strains was quantified in the presence of different hydrocarbons to confirm the oil displacement activity. The recovery assays included acid precipitation, solvent extraction, ammonium sulfate, and zinc sulfate precipitation methods. All these methods revealed that the amount of biosurfactants correlates to the type of tested hydrocarbons, where the highest amount was produced in crude oil contaminated samples. In conclusion, the study highlights the importance of *Candida* isolated from contaminated soils for bioremediation of petroleum oil pollution. That raises the need for further analyses on the microbes/hydrocarbon degradation dynamics.

## Introduction

Hydrocarbons are a rich energy and carbon source for hydrocarbon-degrading microorganisms. Over 100 fungal genera have been identified as significant oil-degraders^1^. Microbial hydrocarbon degradation involves complex enzymatic activities such as hydroxylases, dehydrogenases, monooxygenases, dioxygenases, oxidoreductases, etc.^2^. Although the different pathways have been extensively examined, there is limited understanding of enzymatic mechanisms and their associated genetic pathways of hydrocarbon degradation in fungi^3^. Fungi facilitate the degradation of recalcitrant hydrocarbons by secreting extracellular enzymes that transform the hydrocarbons into intermediates with lower toxicity^4^.

So far, studies on fungal bioremediation have mostly revolved around terrestrial environments^5^; marine environments, on the other hand, are not very commonly examined^6^. Effective biodegradation of crude oil by marine fungi was determined by quantifying the changes in the total mass of crude oil over time^6^. It has also been reported that fungi isolated from hydrocarbon-contaminated habitats in the Gulf of Mexico can degrade n-alkanes and polycyclic aromatic hydrocarbons^7^. Additionally, some fungi can facilitate hydrocarbon bioavailability to other microbial communities, such as other bacteria or fungi, by biosurfactants production^8^.

The most significant characteristic of a potential hydrocarbon degrader is the ability to produce biosurfactants via microbes^8^. The biosurfactants cause oily contaminants to become more soluble, which increases their availability as carbon sources for microorganisms and further increases their degradation^9^. The properties of microbial surfactants are analogous to synthetic surfactants, though the former is naturally biodegradable and can be produced in situ^10^. Isolation of microorganisms with specific features to emulsify and solubilize hydrophobic contaminants both ex-situ and in situ is a significant advantage over competitors in contaminated environments^8^. These processes involve directly implementing microbes or microbial surfactants in the contaminated wells, which assists in reducing oil viscosity and leads to unobstructed flow through the pipelines and more stabilized fuel water–oil emulsions^11^.

The current study aimed to isolate and recognize yeast communities found in chronically hydrocarbon-contaminated petrol stations in Riyadh, Saudi Arabia. These isolates were examined for their ability to use crude oil as a distinct carbon source and to investigate their ability to create biosurfactants.

## Results

### Identification of *Candida* strains in different Soil samples:

In the current study, six soil samples were collected from three different spots (two/each) surrounding the crude oil reservoirs et al. Faisaliyyah, Al Sina’iyah, and Ghubairah in the Riyadh region of Saudi Arabia. The physical characterizations showed that the soil samples had different colors and pH degrees. The soil samples from Al Faisaliyyah had umber-brown color with acidic pH of 5.89, those from Al Sina’iyah had caramel-brown with a pH of 7.72, while those from Ghubairah had a Mocha-brown color with a pH of 7.64. The Potato Dextrose Agar (PDA) cultures revealed the presence of 4 strains of *Candida* species with an incidence of 13.33% in all soil samples. The yeast species were identified using standard taxonomic keys based on typical mycelia growth and morphological characteristics provided in the mycological keys. Based on the physical and microscopic diagnosis, the isolated species were identified as *Candida parapsilosis, Candida krusei, Candida famata, *and *Rhodotorula* spp.

**Figure 1:**
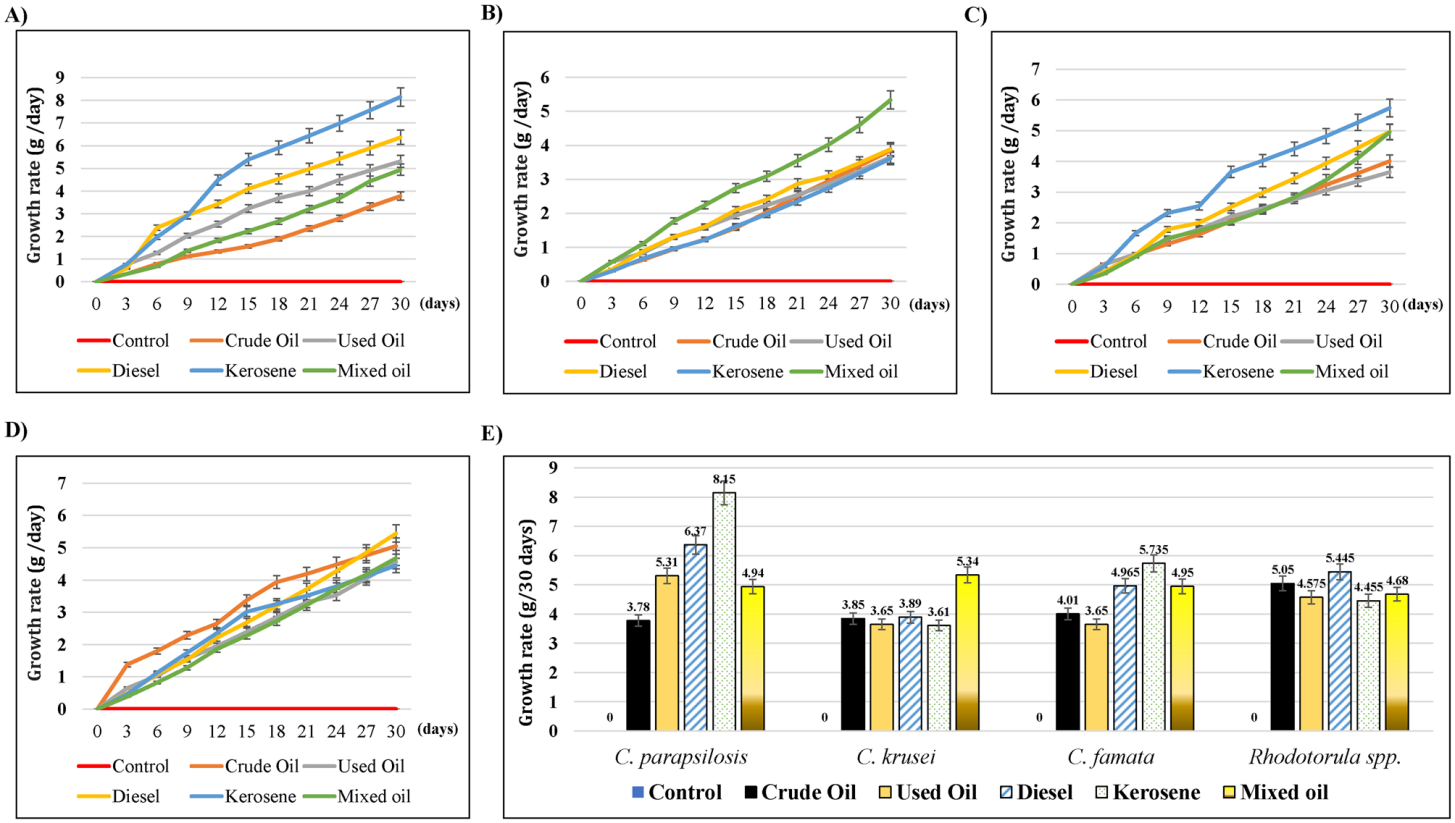
Effect of different Hydrocarbons on the growth rate of soil-isolated strains. The yeast strains were grown on MSM liquid medium mixed with 1% of either crude oil, used oil, diesel, kerosene, or mixed oil. The growth was observed for 30 days. The statistical analysis of differences in growth rates of the isolated strains was performed using SPSS statistical package (version22) for the *One-way ANOVA* and Dunnett's, where the values were significant *P* < 0.05*.* (**A**) *C. parapsilosis*, (**B**) *C. krusei, *(**C**) *C. famata, *and (**D**) *Rhodotorula* spp. (**E**) Bar chart of the growth rate of all the isolated strains, 30 days post-incubation with different hydrocarbons.

We calculate the growth rate of the isolated strains to test the hydrocarbon tolerance for 30 days (Supplementary Figure 1). The growth rate of tested fungi differed depending on the carbon source used. That was evidenced by the change of color in each flask. As shown in Fig. 1A, the growth rate of *C. parapsilosis* increased through the 30 days, while kerosene induced the highest significant growth rate (8.15 g/ 30 days, *P* = *0.02*), followed by diesel oil (6.37 g/30 days, *P* = *0.18*), used oil (5.31 g/30 days, *P* = *0.43*), and mixed oil (4.94 g/30 days, *P* = *0.55*), as compared to the lowest growth rate induced by crude oil (3.78 g/30 days). In the cultures of *C. krusei* (Fig. 1B), kerosene induced a non-significant increase in its growth rate (5.34 g/30 days), whereas other hydrocarbons caused similar growth rates (3.61–3.89 g/30 days), as compared to the crude oil (3.85 g/ 30 days). Similar to *C. parapsilosis, *kerosene induced the highest increase in the growth of *C. famata* (5.73 g/ 30 days), followed by Diesel oil (4.97 g/ 30 days), mixed oil (4.95 g/ 30 days), used oil (3.65 g/ 30 days), as compared to the crude oil treatment (4.01 g/ 30 days) (Fig. 1C), despite all of them were non-significant. Finally, there were non-significant changes in the growth rate of *Rhodotorula* spp., (Fig. 1D) in the presence of different tested hydrocarbons. However, Diesel induced the highest growth rate by 5.45 g/ 30 days. The growth rates of different strains at 30 days were blotted together to compare the tolerance to the tested hydrocarbons (Fig. 1E). From another perspective, the comparison between different tested hydrocarbons revealed that *C. parapsilosis* was the highest consumer for the carbon sources in Kerosene, diesel, and the used oil, as it’s clear from the highest growth rates (Fig. 1E). Similarly, *C. krusei* and *Rhodotorula* spp. had the highest growth rates for the treatments with mixed and crude oils, respectively. That suggested that the type of hydrocarbon might affect the growth rate of specific species. All strains had higher significant growth rates against the untreated control *(P* < *0.001).*

### Different morphological changes induced after treatments with crude oil

**Figure 2:**
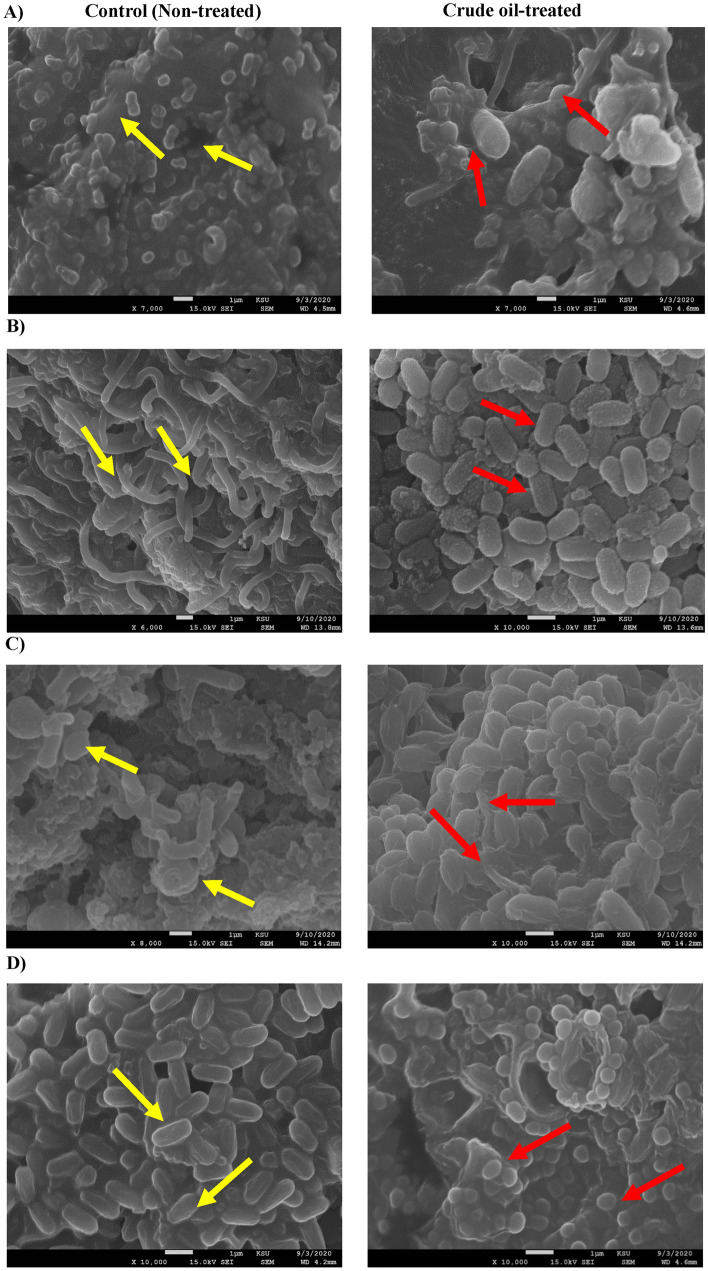
Morphological changes induced by crude oil treatment. SEM images of tested strains with and without 1% of crude oil. Yellow arrows indicated the morphology of untreated control samples, where red arrows denoted the structural changes induced by the crude oil treatment. (**A**) C*. parapsilosis*, (**B**) *C. krusei,* (**C**) *C. famata*, and (**D**) *Rhodotorula* spp.

The SEM results of *C. parapsilosis* revealed different morphological changes in the outer surfaces of *Candida* strains post-treatment with 1% crude oil as compared to the untreated control (Fig. 2A). The SEM images of the untreated cells had a natural structure with smooth flat surfaces, while the treated cells had large unequal sizes with the unusual zigzag surface structures. In *C. krusei*, the untreated samples were similar to smooth saprophytes, while the crude oil treatments deformities the cellular surface into an oval shape with a grainy and sinuous structure (Fig. 2B). Similarly, post-treatment of *C. famata* induced an oval cellular shape with smooth edges and abnormal coatings, while the control cells were similar to the smooth-shape shoot plants (Fig. 2C). Finally, the SEM screening of *Rhodotorula* species revealed the ability of crude oil to induce some cellular changes, where the cells appear as if they were surrounded by an extra membrane (Fig. 2D).

### *Candida* spp. induced biodegrading of different hydrocarbons

**Figure 3:**
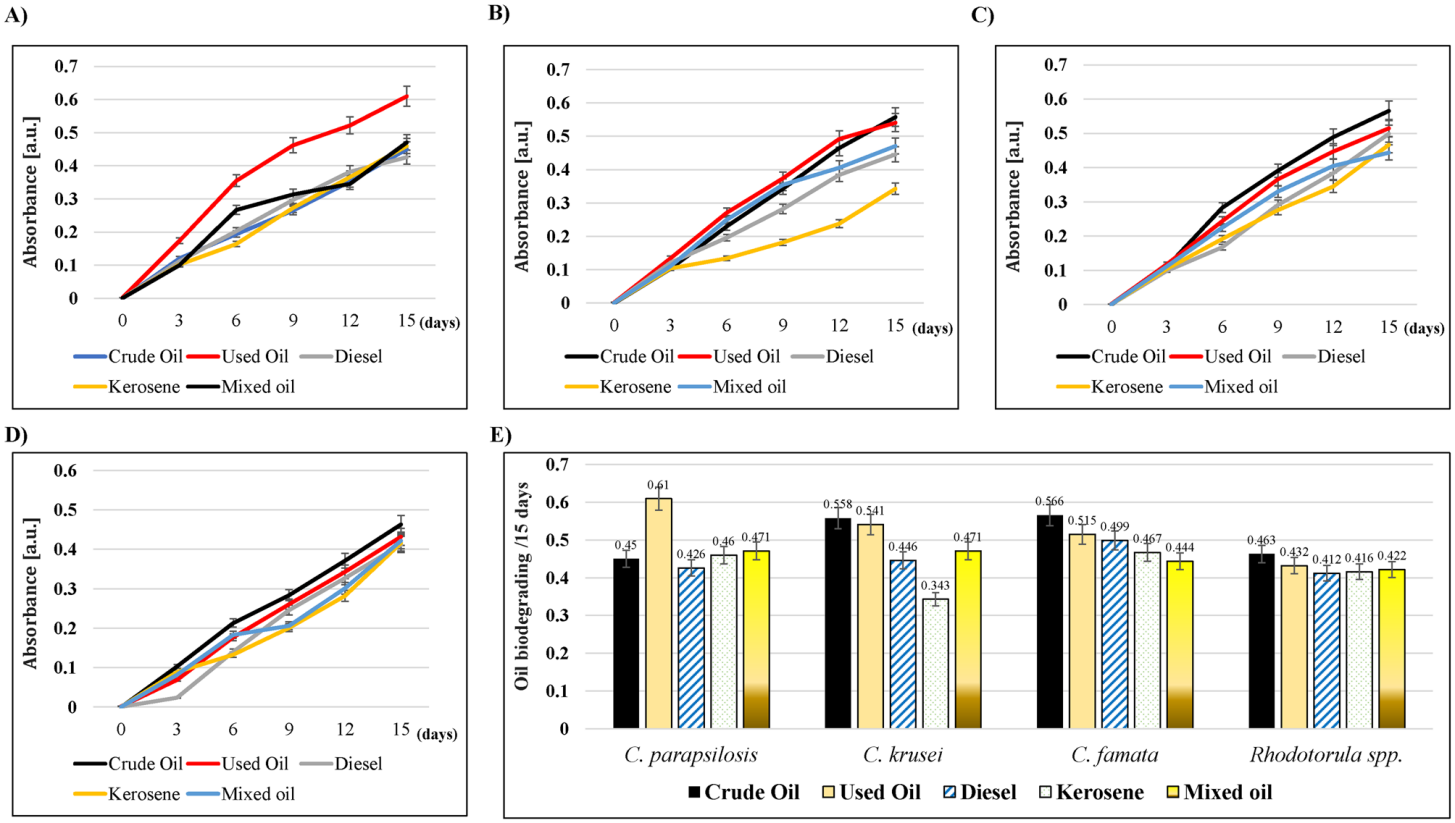
The oil biodegrading ability of different yeast isolates on different hydrocarbons. The strains were grown in MSM supplemented with 0.1% Tween 80, and 0.6 mg/ml DCPIP, incubated at 25 °C for 15 days, then the colorimetric changes in DCPIP were measured at 420 nm. (**A**) *C. parapsilosis,* (**B**) *C. krusei,* (**C**) *C. famata*, and (**D**) *Rhodotorula* spp. (**E**) Bar chart of the growth rate of all the isolated strains, 15 days post-incubation with different hydrocarbons.

Different strains of candida were tested for their ability to oxidize the oil hydrocarbons by interacting with the redox dye (2, 6-dichlorophenol indophenol (DCPIP)). That allowed the transfer of electrons to DCPIP, which changed its color from blue to colorless^12^. In the current study, mixing different hydrocarbons with DCPIP didn’t induce any oxidation; however, it produced a light violet color (Supplementary Figure 2A). Otherwise, treatment with different Candida strains caused the oxidation of oils. As shown in Fig. 3A, *C. parapsilosis* induced the oxidation of all oils. The highest effect was for used oil (0.61 a.u.), whereas the least was for Diesel (0.426 a.u.) on the 15th-day post-treatment. Treatment with *C. krusei* showed a similar effect on all tested hydrocarbons; however, the lowest oxidation was for kerosene (0.343 a.u.) on the 15th day of incubation (Fig. 3B). For *C. famata* and *Rhodotorula* spp., there were no differences between all tested hydrocarbons (Fig. 3C); however, they caused the discoloring of DCPIP (Supplementary Figure 2B–E). The comparison of all tested organisms shown in Fig. 3E revealed that crude oil was more sensitive to the oxidation induced by the treatment with *C. famata* (0.556 a.u.) and *C. krusei* (0.558 a.u.) on the 15th day of incubation. *C. parapsilosis* was the strongest bio-degrader of kerosene (0.46 a.u.) and mixed oil (0.471 a.u.). Finally, the used and diesel oils were more oxidized by *C. parapsilosis* (0.61 a.u.) and *C. famata* (0.499 a.u.), respectively. *Rhodotorula* spp. was the weakest oxidizer among all isolates, which was represented by the reddish-brownish colors of different hydrocarbons that indicated the incomplete reduction of DCPIP (Supplementary Figure 2E).

### *Candida* strains affected the physical properties of tested hydrocarbons

The results showed that the products of the isolated *candida* strains acted as biosurfactants of the tested hydrocarbons. That caused the collapse of all oil drops (Table 1). The results showed that the highest collapsing effect occurred with crude, used, and mixed oils. Furthermore, diesel and kerosene were the lowest affected hydrocarbons compared to positive and negative controls.

**Table 1:** Drop collapse assay for surfactants of the isolated strains of yeast.

Microorganism	Hydrocarbon source						
	− C	+ C	Crude oil	Used oil	Diesel	Kerosene	Mixed oil
*C. parapsilosis*	–	+++	+++	+++	+	+	+++
*C. krusei*	–	+++	++	++	+	+	+++
*C. famata*	–	+++	++	++	+	+	++
*Rhodotorula spp.*	–	+++	++	++	+	+	++

− C: negative control (culture broth), + C: positive control (Triton X-100).

**Figure 4:**
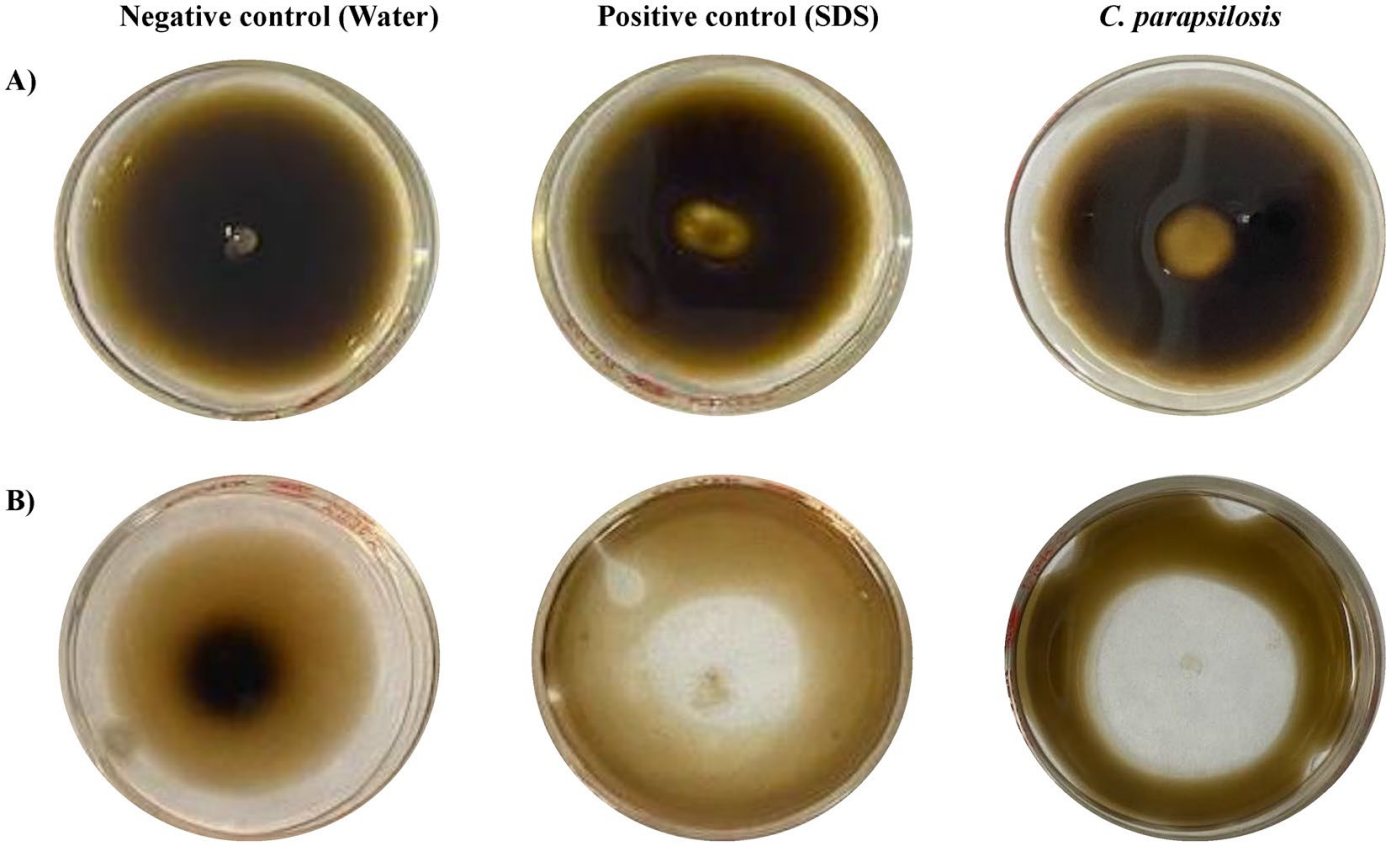
Oil spreading assay of *C. parapsilosis*. An amount of 20 ml of water was added to the Petri plate (100 mm) followed by 20 µl of either crude oil (**A**) or mixed oil (**B**), which formed a thin layer on the surface of the water. Then, 10 µl of either water, 1% SDS, or CFSs of *C. parapsilosis* were added to the surface and the spreading of the oil was noticed.

In the current study, CFS of *C. parapsilosis* formed a clear zone of 9.7 ± 1.1 mm diameter over the surface of crude oil, which was greater than that of Sodium Dodecyl Sulfate (SDS) with a zone diameter of 7.7 ± 0.8 mm (Fig. 4A). For mixed oil, *C. parapsilosis* formed a larger zone of 31.9 ± 0.15 mm diameter, which was greater than the SDS zone with a diameter of 20.3 ± 0.1 mm (Fig. 4B). The negative control of distilled water didn’t induce any clear zones over the surfaces of the tested oils.

**Figure 5:**
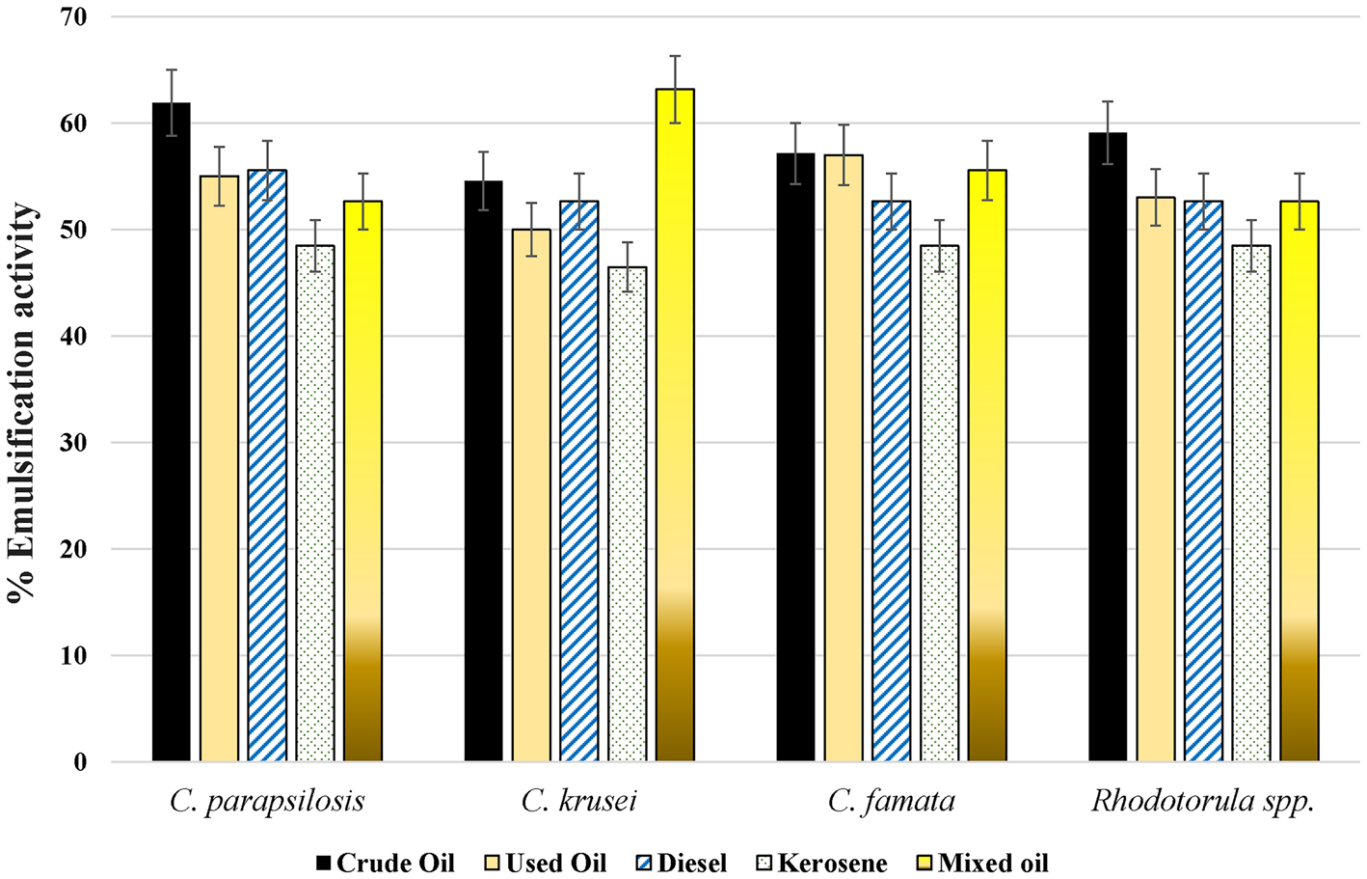
The emulsification activity effects of different yeast strains on tested hydrocarbons. CFSs of isolated strains were mixed with the tested hydrocarbons and incubated for 24 h at 25 °C. The percentage of emulsification was calculated by measuring the percentage of the higher emulsified layer to the total height of the mixture.

Furthermore, the isolated strains showed appropriate bio-emulsification activity against all tested oils (Supplementary Figure 3). All treated samples showed the formation of two separate layers, which were different in their heights according to the type of oil and treatment. As shown in Fig. 5, *C. parapsilosis* was the strongest microbial bio-emulsifier of crude oil (61.9%), followed by *Rhodotorula* spp. (59.09%), *C. famata* (57.14%), and *C. krusei* (54.54%). For the used oil samples, *C. famata* was the strongest bio-emulsifier (57%), followed by *C. parapsilosis* (55%), *Rhodotorula spp. *(53%), and *C. krusei* (50%). In Diesel-treated samples, *C. parapsilosis* was the strongest bio-emulsifier at 55.55%, whereas the other three organisms had the same bio-emulsification activity of 52.63%. For kerosene, *C. parapsilosis, Rhodotorula* spp., and *C. famata* had bio-emulsification activity of 48.48%, while *C. krusei* had lower activity of 46.48%. Unlike kerosene, *C. krusei* exhibited strong emulsification of mixed oil by 63.15%, followed by *C. famata* (55.55%), *C. parapsilosis, *and *Rhodotorula* spp. (52.63%).

### The amount of biosurfactants recovered from *Candida* strains correlates to the type of tested hydrocarbons

**Figure 6:**
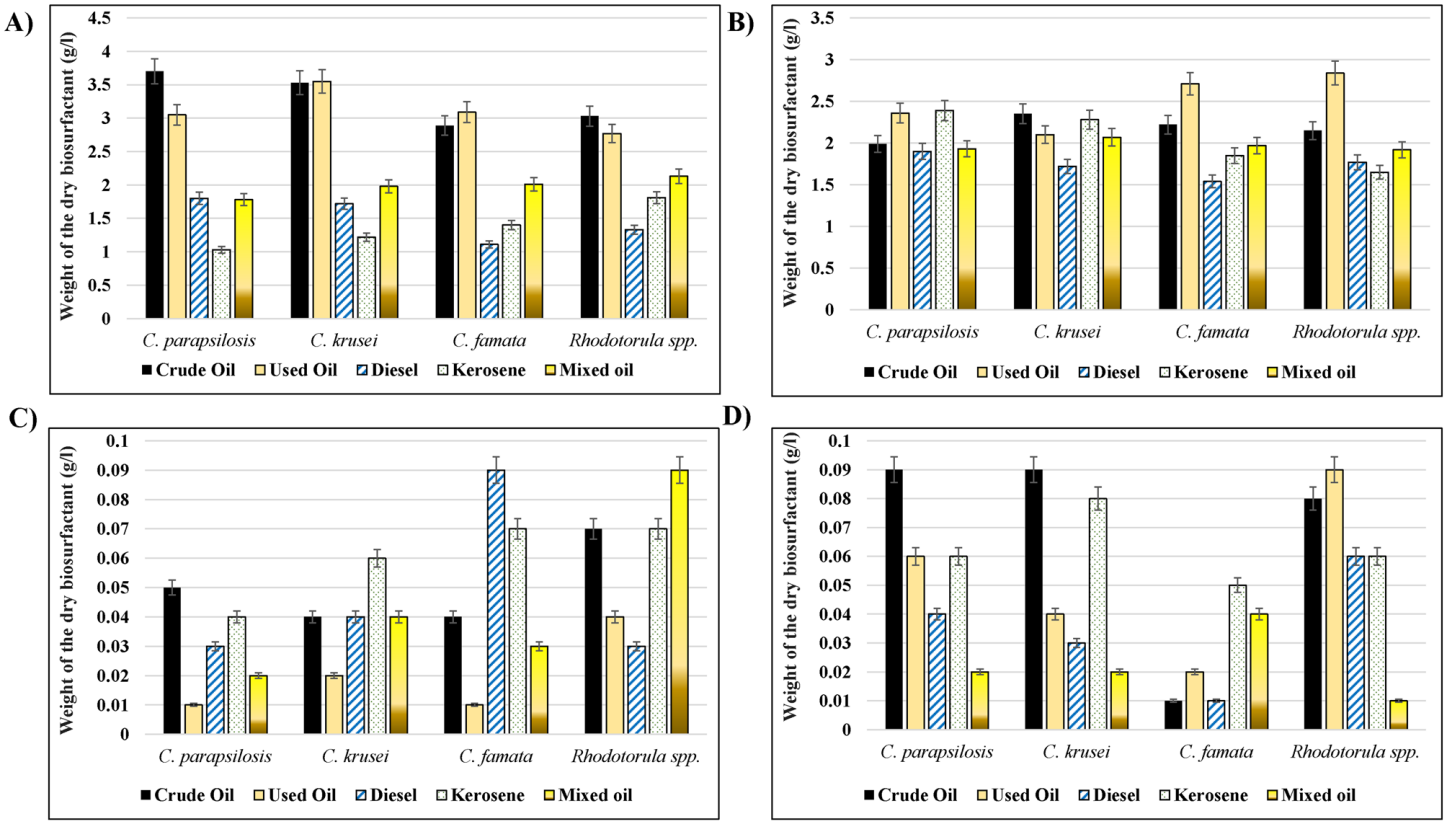
Biosurfactants recovery assays. The amount of biosurfactants produced by different isolated strains growing in the presence of different hydrocarbons were isolated and measured by different recovery assays. (**A**) Acid precipitation, (**B**) Solvent Extraction, (**C**) Ammonium Sulfate, (**D**) Zinc Sulfate.

To confirm the oil displacement activity of the isolated Candida strains, the content of produced biosurfactants was tested and compared in the presence of different hydrocarbons. The precipitation with HCl resulted in white color powder in untreated samples, where the treated samples produced slightly yellowish color precipitates, data not shown. As shown in Fig. 6A, the most productive biosurfactant in *C. parapsilosis* and *C. krusei* were produced with crude oil (3.7 g, 3.55 g), where the lowest amount was for kerosene (1.03 g, 1.22 g), respectively. Similarly, *Rhodotorula* spp. produced the highest amount of biosurfactant with crude oil (3.03); however, the lowest amount being for Diesel (1.33 g). In *C. famata*, the highest production of biosurfactants was for used oil (3.09 g), while Diesel had the least amount (1.11 g). In comparison among different organisms, the highest content of biosurfactants was produced by *C. parapsilosis* for crude and diesel oils, *C. krusei* for used oil, *Rhodotorula* spp. for kerosene, and mixed oil, where *C. famata* was the lowest producer with all hydrocarbons.

The solvent extraction assay was used to differentiate between the soluble (Biosurfactants) and insoluble constituents (non-emulsified hydrocarbons and microbial cells), which resulted from the reaction between isolated microbes and tested hydrocarbons (Fig. 6B). The highest amount of the dry-weight white precipitate resulted from reactions between *Rhodotorula* spp. (2.86 g) or *C. famata* (2.71) with used oil, *C. parapsilosis* with kerosene (2.39 g), and *C. krusei* with crude oil (2.35 g). Diesel and mixed oil treatments with different isolates resulted in almost smaller amounts of precipitate by comparing to other hydrocarbons.

Finally, another two methods were used to test the biosurfactant recovery of tested microbes, the Ammonium sulfate, and Zinc sulfate precipitation methods. As shown in Fig. 6C, the used oil resulted in the least precipitation of Ammonium sulfate when treated with *C. famata* (100 mg)*, C. parapsilosis* (100 mg)*, *and *C. krusei* (200 mg). Diesel oil resulted in the least precipitation with *Rhodotorula* spp. (300 mg). The dry weight of the precipitate produced in the Zinc sulfate precipitation method (Fig. 6D) showed that the lowest biosurfactant production resulted from the reaction of mixed oil with *Rhodotorula* spp. (100 mg), *C. parapsilosis* (100 mg)*, *and *C. krusei* (100 mg). In the case of *C. famata, *both diesel and crude oil produced the lowest biosurfactant amount (100 mg)*.*

## Discussion

The sensitivity of different microorganisms to environmental changes can affect their viability or induce biodegradation^13,14^. Different factors control the microbial biodegradation rate of hydrocarbons, such as their type, availability, length, volatilization, and solubility, which act as sources of nitrogen^15,16^. Other environmental factors such as pH, temperature, humidity, salinity, oxygen availability, and nutrient content might affect the existence of different microbes^13,17^. In the current study, pH and hydrocarbons content of soil samples from different crude oil reservoirs allowed the growth and existence of four yeast strains; *C. parapsilosis, C. krusei, C. famata, *and *Rhodotorula* spp. A previous study indicated that the lower pH of soil samples resulted from the higher alkalinity due to crude oil carbonaceous constituents, which further allowed the microbial growth in these contaminated soils^18^. The fact that they were isolated from contaminated soil samples shows that the contamination did not inhibit the growth and variation of fungal strains in these polluted environments. That also demonstrates that the fungal species used oil compounds as nutrients, where the crude oil pollution caused an increase in fungal growth^19^. All isolates were examined for their ability to grow to utilize various carbon sources such as crude oil, kerosene, used (engine) oil, diesel, or a mixture of these oils as a unique carbon source.

The results revealed the ability of these organisms to grow at a 1% concentration on liquid Mineral Salt Medium (MSM), which was significantly higher than the negative controls. That indicated the fungal inability to grow using MSM media as a carbon source. Furthermore, the results revealed that kerosene or so-called ‘paraffin oil’ gave the highest growth rates of all tested species, in contrast to crude oil, which induced the lowest growth. A similar study from Brazil tested the growth rates of some isolated yeast strains, *Meyerozyma guilliermondii*, and *Rhodosporidium diobovatum*, which were the highest in a medium supplemented with kerosene where the growth rate with crude oil was lower^20^. Another study showed that some yeast species, *Candida tropicalis, Candida rugosa, Trichosporon asahii, *and *Rhodotorula mucilaginosa,* were bio-degraders of diesel oil^21^. That was due to the production of different enzymes such as NADPH cytochrome c reductase, catalase, and naphthalene dioxygenase^21^. Different studies showed similar findings, which suggested that yeast strains are reliable bio-remediators that can reduce petroleum contamination in different environments^22–25^. In comparison to the controls, these fungi accumulated high biomass in a liquid medium with all petroleum oils.

The rate at which biodegradation occurs hinges on many factors, such as pollutant characteristics^15^, the microorganism characteristics (cell metabolic pathways and morphological changes)^26^, environmental conditions^17^, and the physicochemical properties of the soil such as density, water holding capacity, pH, moisture, and texture^27^. Microorganisms are highly sensitive to changes in their environments and are affected by composition and hydrocarbon sources^28,29^. In the current study, the SEM imaging of tested isolates showed different morphological changes in the presence of 1% crude oil. SEM was used either to confirm the phenotypic characterization of isolated strains or to study the changes in the outer-surface structures accompanied by crude oil treatment. A similar study used *Candida tropicalis* and revealed cellular morphological changes that cause a significant decrease in the cell diameter^30^. That might be due to the bioaccumulation capacities of these strains that could alleviate soil contamination^31^. No studies were found about the morphological changes in the isolated candida strains in contaminated petroleum spots.

The level of biodegradation in hydrocarbon-polluted soils is contingent on specific factors. That included the environmental conditions^17,32^, the bioavailability of contaminants to microorganisms^33^, and the predominant hydrocarbons types^34^. The spectrophotometric analysis of the growth of the tested strains with 1% of each oil evidenced the oil-biodegradation ability. The higher readings demonstrate a higher concentration of fungal cells as there was a higher absorbance measured at the same absorbance wavelength. The ability of isolated strains to oxidize the tested oil hydrocarbons was studied. All isolates were observed to be potent according to the qualitative (DCPIP and spectrophotometry) analysis. A similar study was conducted in the Gulf of Mexico and showed the ability of some fungi to induce crude-oil-degrading that was confirmed by decolorization of DCPIP, reduction in the quantity of crude oil, and fungal proliferation^35^. Another study showed that the strains of *Candida tropicalis*, *Rhodotorula mucilaginosa*, and *Rhodosporidium toruloides* isolated from the Khafji oil field, Saudi Arabia showed a crude oil biodegradable activity, which induced the decolorization of DCPIP dye^36^. Another study revealed the ability of *Candida viswanathii* to biodegrade biodiesel, which caused the decolorization of the DCPIP redox dye^37^. In a study from Pernambuco, Brazil, *Rhodotorula aurantiaca* and *Candida ernobii* isolated from petroleum-contaminated soil samples induced biodegradation of diesel oil^38^. That caused lower O.D. values due to the decolorization of DCPIP^38^. All these studies provided evidence about the ability of the isolated strains to oxidize the carbon source, which induced the electronic transfer to DCPIP and resulted in its decolorization^36^. Furthermore, that evidenced the capacity of isolated strains to degrade crude oil.

Detection of biosurfactant-producing fungi was assessed by drop-collapsing, oil-spreading, and emulsification activities as sensitive and rapid methods. Drop collapse assay is one of the techniques used to measure the destabilization of liquid droplets by surfactants, which prevent the repel of the polar water molecules from the hydrophobic surface^37^. In contrast, the presence of surfactants allows the spread/collapse of the drops due to the reduction of the interfacial tension^38^. One of the most characteristic features of aromatic oils is the ability of different biosurfactants to form clear zones over the oil surface^39^. The diameter of this zone correlates to the oil displacement activity^40^. The presence of biosurfactants in a supernatant leads to the formation of a halo which can be measured and compared to the positive and negative controls^41^.

In the current study, all isolates caused the collapse of all oil drops from different hydrocarbon sources. Further, the drop-collapsing effect varied according to the type of hydrocarbon source. Pure filtered oils, diesel, and kerosene had the highest drop-collapsing effect. Besides, the current study demonstrated the ability of *C. parapsilosis* to form clear zones over the surfaces of crude and mixed oils. The emulsification activities of crude oil, used oil, diesel, kerosene, and mixed oil ranged from 41 to 61%, whereas the lowest emulsification activity for the yeast strains was seen for kerosene. That evidenced its ability to change the physical properties of these oils by increasing the oil spreading^41^. In agreement with our findings, a previous study illustrated the biosurfactant produced by *C. parapsilosis* was positive for oil spreading assay, drop-collapse method, and emulsifying index, despite it being negative for hemolytic activity in the blood agar^42^. Similar studies showed the oil spreading and emulsification activities of *Candida glabrata* to n-hexadecane^43^, *Rhodotorula babjevae* to the crude oil at 38.46 mm^2^^44^, and *C. tropicalis* and *C. bombicola* to waste frying oil^45,46^. All these studies reported that the biosurfactants produced by yeast strains might affect hydrocarbon bioavailability and biodegradation.

Most of the fungi utilize petroleum hydrocarbons, as a source of carbon and energy, and metabolize the molecules to CO_2_ and biomass^47^. The chemical composition of different hydrocarbons is an important factor that determines the ability of fungal growth^48^. The oil displacement area in the oil spreading test was directly proportional to the concentration of biosurfactants in the solution^49^. In the current study, four different recovery methods were employed to measure the amounts of biosurfactant produced by the studied strains in the presence of different hydrocarbons. according to the above studies, the current results showed that the amount of biosurfactant depends on the type of hydrocarbon and the extraction method used. The highest amount of biosurfactant from *C. parapsilosis* and *Rhodotorula* spp. were produced by crude oil by using the acid precipitation method. In *C. krusei* and *C. famata, *used oil was the highest producer of biosurfactants according to the acid precipitation method, as well. In the solvent extraction method, used oil showed the highest amount of biosurfactants produced from *C. parapsilosis,* *C. famata, *and *Rhodotorula* spp., while crude oil showed the highest production with *C. krusei.* The differentiation in the biosurfactant yields might be due to the hydrophobic end which increased their solubility in an organic solvent^50^.

A previous study suggested that most biosurfactants are synthesized in media containing carbon sources (e.g., carbohydrates, fats, oils, hydrocarbons) by aerobic microorganisms^51^. These biosurfactants are amphipathic compounds, which possess both hydrophobic and hydrophilic moieties and exhibit various amphiphilic structures^52^. Following our findings, previous studies showed that biosurfactants’ recovery depended mainly on their ionic charge and solubility in the desired solvent, which might explain the different yields produced by isolated strains^8,50,52^.

Minor amounts of biosurfactants were produced by the ammonium sulfate and zinc sulfate methods. On the other hand, ionic precipitation had almost the same number of yields. That might be because the emulsification activity of an organism depends on the pH and divalent cations such as magnesium ions^52^. In agreement with our findings, the amount of rhamnolipid biosurfactant produced by *Pseudomonas aeruginosa* was higher when produced by organic solvent extraction (7.37 ± 0.81 g/L), which was higher than the amount produced by zinc sulfate precipitation (5.83 ± 0.02 g/L)^53^.

The current study revealed that fungi isolated from soils contaminated with petroleum products appear as a promising microbial resource for bioremediation of crude oil pollution. To our knowledge, the isolated species were not detected before in the contaminated soil samples from the oil reservoirs in the Riyadh region, Saudi Arabia. Besides, the oil biodegradation capability of *Candida famata,* and *Rhodotorula* spp. was not fully tested before as shown in the current study. That raises the need for further analyses on the most promising isolates to accurately determine the kind of hydrocarbons that are metabolized and the degradation dynamics. Besides, the study highlights the importance of intraspecific variability. That emphasizes the relevance of high-throughput culturing strategies to obtain different microbial isolates. That was coupled with high-throughput screening approaches to efficiently determine the most promising isolates. Those isolates can efficiently utilize hydrocarbons and produce biosurfactants. So, *Candida* can be useful for bioremediation applications within the frame of bioaugmentation or bio-stimulation processes. Further studies will be required to identify the exact components of the biosurfactants produced by these species. Furthermore, more studies are required to assess the cellular changes induced by various enzymatic pathways involved in microbial oil-biodegradation.

## Materials and methods

### Soil sample collection

Soil samples were collected from three different crude oil reservoirs et al. Faisaliyyah, Al Sina’iyah, and Ghubairah located in Riyadh, Saudi Arabia. Briefly, 400 g of soil samples were collected at 0–10 cm depth, under aseptic conditions. Samples were sieved by 2.5 mm pore size sieves, homogenized, and stored at 4ºC until use.

### Sources of different hydrocarbons

Different samples of crude oil, kerosene, diesel, and used oil were collected in sterile flasks from the tankers of Saudi Aramco Company (Dammam, Saudi Arabia). Additionally, another flask was prepared by mixing 1% of each oil in MSM liquid media to make up the mixed oil. The oil samples were sterilized by Millex® Syringe Filters (Merck Millipore co., Burlington, MA, United States) and stored at 4 °C for further usage.

### Isolation and identification of fungal species

The fungal species in the soil contaminated by crude oil were identified using the dilution method. Briefly, 10% of each soil sample was dissolved in distilled water and vortexed thoroughly. Then, 0.2 ml of each sample was cultured on a sterile PDA plate incubated at 28 °C for three days until the growth of different fungal colonies. Carefully, each colony was isolated, re-cultured on new PDA McCartney bottles of PDA slant, and incubated at 28 °C for three days. The fungi were identified microscopically using standard taxonomic keys based on typical mycelia growth and morphological characteristics provided in the mycological keys^54^. Besides, the taxonomy of the isolated yeast strains was confirmed by the API 20 C AUX kit (Biomerieux Corp., Marcy-l'Étoile, France) (data not shown). The morphology of pure cultures was tested and identified under a light microscope as described before^55^.

The incidence of each strain was calculated as follows:

$$ Incidence \;(\% ) = \frac{{{\text{Number }}\;{\text{of }}\;{\text{samples }}\;{\text{showed }}\;{\text{microbial }}\;{\text{growth}}}}{{{\text{Total }}\;{\text{samples}}}} \times 100 $$

### Hydrocarbon tolerance test

The growth rate of isolated strains was tested in a liquid medium of MSM mixed with 1% of either crude oil, used oil, diesel, kerosene, or mixed oil. Furthermore, a control sample of MSM liquid medium without any of the oils tested and all culture media were autoclaved at 121 °C for 30 min. After cooling, 1 ml of each isolate was inoculated with one of the above mixtures and incubated at 25 °C on an orbital shaker. The growth rate was measured every three days for a month for each treatment versus the control. All experiments were performed in triplicates.

### Scanning electron microscopy (SEM)

The morphology of different strains of the isolated fungi was tested by SEM, as previously described^56^, with some modifications. Briefly, 1 ml of each growing strain, in the liquid media, was centrifuged at the maximum speed (14,000 rpm) for 1 min, followed by fixation with 2.5% glutaraldehyde, and overnight incubation at 5 °C. Later, the sample was pelleted, washed with distilled water, then dehydrated with different ascending concentrations of ethanol (30, 50, 70, 90, 100 (v/v)) for 15 min at room temperature. Finally, samples were examined in the Prince Naif Research Centre (King Saud University, Riyadh, Saudi Arabia) by the JEOL JEM-2100 microscope (JEOL, Peabody, MA, United States), according to the manufacturer instructions.

### Crude oil degradation assay

A modified version of the DCPIP assay^57^ was employed to assess the oil-degrading ability of the fungal isolates. For each strain, 100 ml of the autoclaved MSM was mixed with 1% (V/V) of one of the hydrocarbons (crude oil, used oil, diesel, kerosene, or mixed oil), 0.1% (v/v) of Tween 80, and 0.6 mg/mL of the redox indicator (DCPIP). Then, 1–2 ml of different fungi growing in liquid media (24–48 h) add to the Crude Oil Degradation media, prepared previously, and incubated for two weeks in a shaking incubator at 25 °C. All flasks were covered and protected from light, aeration, or temperature exchanges to reduce the effects of oil weathering (evaporation, photooxidation). The surfactant Tween 80 was used for bio-stimulation and acceleration of the biosurfactant production by increasing metabolism^58^. A non-inoculated Crude Oil Degradation media was used as the negative control. Afterward, the colorimetric analysis for the change in DCPIP color was estimated, spectrophotometrically, at 420 nm. All experiments were performed in triplicates.

### Preparation of cell-free supernatant (CFS)

To prepare the Cell-Free Supernatant (CFS), all isolates were grown in MSM broth medium with 1% of either crude oil, used oil, diesel, kerosene, or mixed oil for 30 days in a shaking incubator at 25 °C. After incubation, the cells were removed by centrifugation at 10,000 rpm for 30 min at 4 °C. The supernatant (CFS) was collected and filter-sterilized with a 0.45 μm pore size sterile membrane. CFS was screened for the production of different biosurfactants. All the experiments were carried out in triplicates, and the average values were calculated.

### Drop-Collapse assay

The Drop-Collapse assay was performed as previously described^9^, with some modifications. 100 µl of crude oil was applied on glass slides, then 10 µl of each CFS was added to the center of the slide surface and incubated for a minute at room temperature. The slides were imaged by a light microscope using the 10X objective lenses. The spreading on the soil surface was scored by either « + » to indicate the level of positive spreading, biosurfactant production, or «—» for negative spreading. Biosurfactant production was considered positive at the drop diameter ≥ 0.5 mm, compared to the negative control (treated with distilled water).

### Oil spreading assay

An amount of 20 ml of water was added to the Petri plate (size of 100 mm) and mixed with 20 µl of crude oil or mixed oil, which created a thin layer on the water surface. Then, 10 µl of CFS was delivered onto the surface of the oil, and the clear zone surrounding the CFS drop was observed. The results were compared to the negative control (without CFS) and positive control of 1% SDS^41^. We have measured the clear zones diameter from images and calculate the actual values in regards to the diameter of the Petri dish (10 cm). The assay was performed in triplicates.

### Emulsification activity assay

The emulsification activity of each isolate was assessed by mixing equal volumes of MSM broth medium of each isolate with different oils in separate tubes. The samples were homogenized by vortex at high speed for two minutes at room temperature (25 °C) and allowed to settle for 24 h. The tests were performed in duplicate. Then, the emulsification index was calculated as follows^59^:

$$ Emulsification\; activity\; \left( \% \right) = \frac{{{\text{Height }}\;{\text{of }}\;{\text{emulsion }}\;{\text{layer}}}}{{{\text{Total }}\;{\text{height}}}} \times 100 $$

### Recovery of biosurfactants

The recovery of biosurfactants from CFS was tested through different assays:

### Acid precipitation assay

3 ml of each CFS was adjusted by 6 N HCl to pH 2 and incubated for 24 h at 4 °C. Later, equal volumes of chloroform/methanol mixture (2:1 v/v) were added to each tube, vortexed, and incubated overnight at room temperature. Afterward, the samples were centrifuged for 30 min at 10,000 rpm (4 °C), the precipitate (Light brown colored paste) was air-dried in a fume hood, and weighed^53^.

### Solvent extraction assay

The CFS containing biosurfactant was treated with a mixture of extraction solvents (equal volumes of methanol, chloroform, and acetone). Then, the new mixture was incubated in a shaking incubator at 200 rpm, 30 °C for 5 h. The precipitate was separated into two layers, in which the lower layer (White) was isolated, dried, weighed, and stored^60^.

### Ammonium sulfate precipitation assay

The CFS containing biosurfactant was precipitated with 40% (w/v) ammonium sulfate and incubated overnight at 4 °C. The samples were centrifuged at 10,000 rpm for 30 min (4 °C). The precipitate was collected and extracted with an amount of acetone equal to the volume of the supernatant. After centrifugation, the precipitate (Creamy-white) was isolated, air-dried in a fume hood, and weighed^53^.

### Zinc sulfate precipitation method

Similarly, 40% (w/v) zinc sulfate was mixed with the CFS containing biosurfactant. Then, the mixture was incubated at 4 °C, overnight. The precipitate (Light Brown) was collected by centrifugation at 10,000 rpm for 30 min (4 °C), air-dried in a fume hood, and weighed^53^.

### Statistical analysis

All experiments were performed in triplicate, and the results were expressed as the mean values ± standard deviation (SD). One-way ANOVA and Dunnett's tests were used to estimate the significance levels at *P* < *0.05*. Statistical analyses were performed using the SPSS statistical package (version 22) (IBM, Armonk, NY, United States).

## Supplementary Information

Supplementary Information 1.

Supplementary Information 2.

Supplementary Information 3.

Supplementary Information 4.

Supplementary Information 5.

## Data Availability

All datasets obtained or studied during this study are incorporated in the manuscript.
