# Supplementary figures and images for: The crude oil biodegradation activity of Candida strains isolated from oil-reservoirs soils in Saudi Arabia
Source: Sci Rep. 2022 Jun 23;12:10708. doi: 10.1038/s41598-022-14836-0 (PMC9226172; doi:10.1038/s41598-022-14836-0)

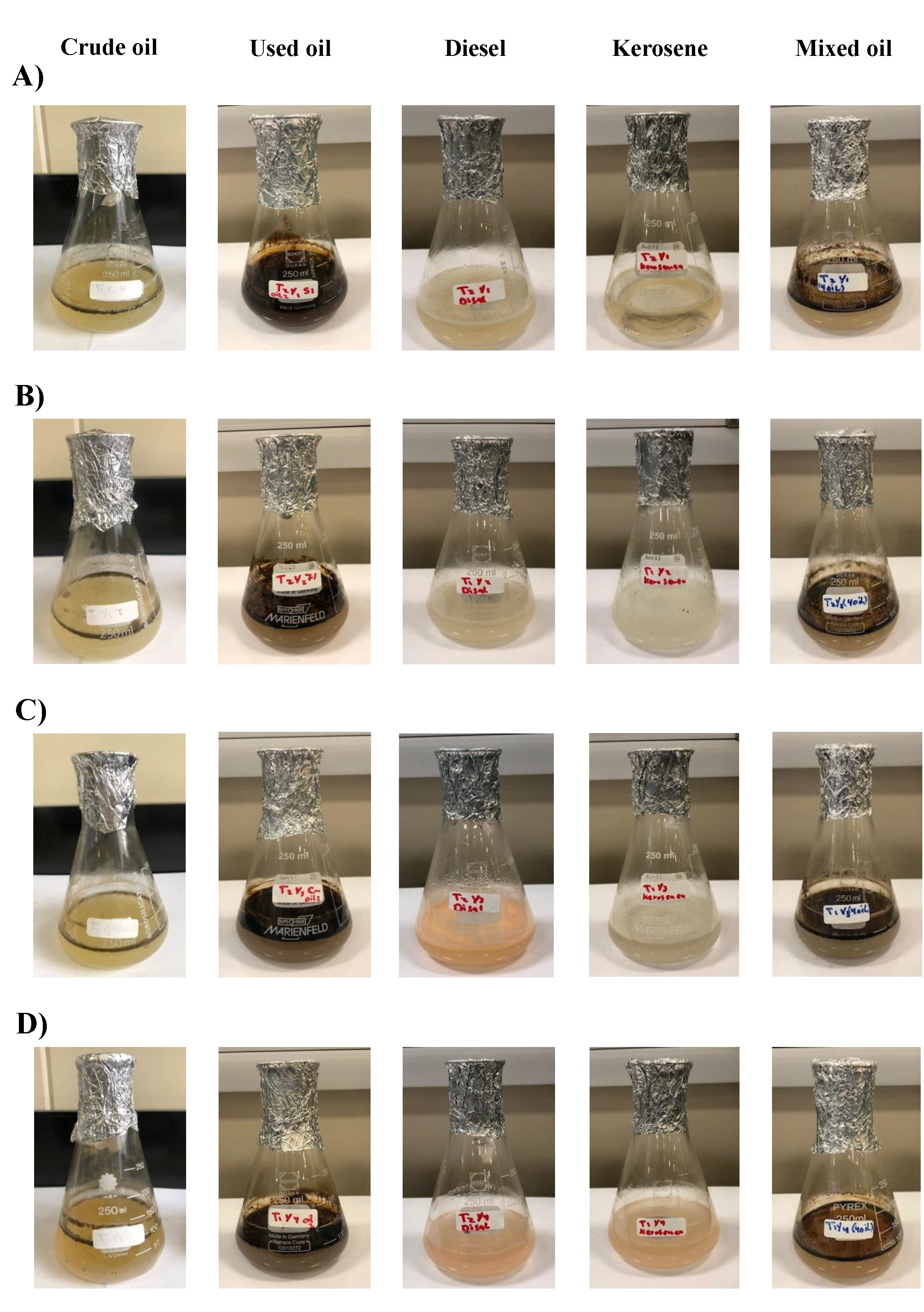

Supplement: Supplementary file 2 — Supplementary Information 2. [file 41598_2022_14836_MOESM2_ESM.tiff]

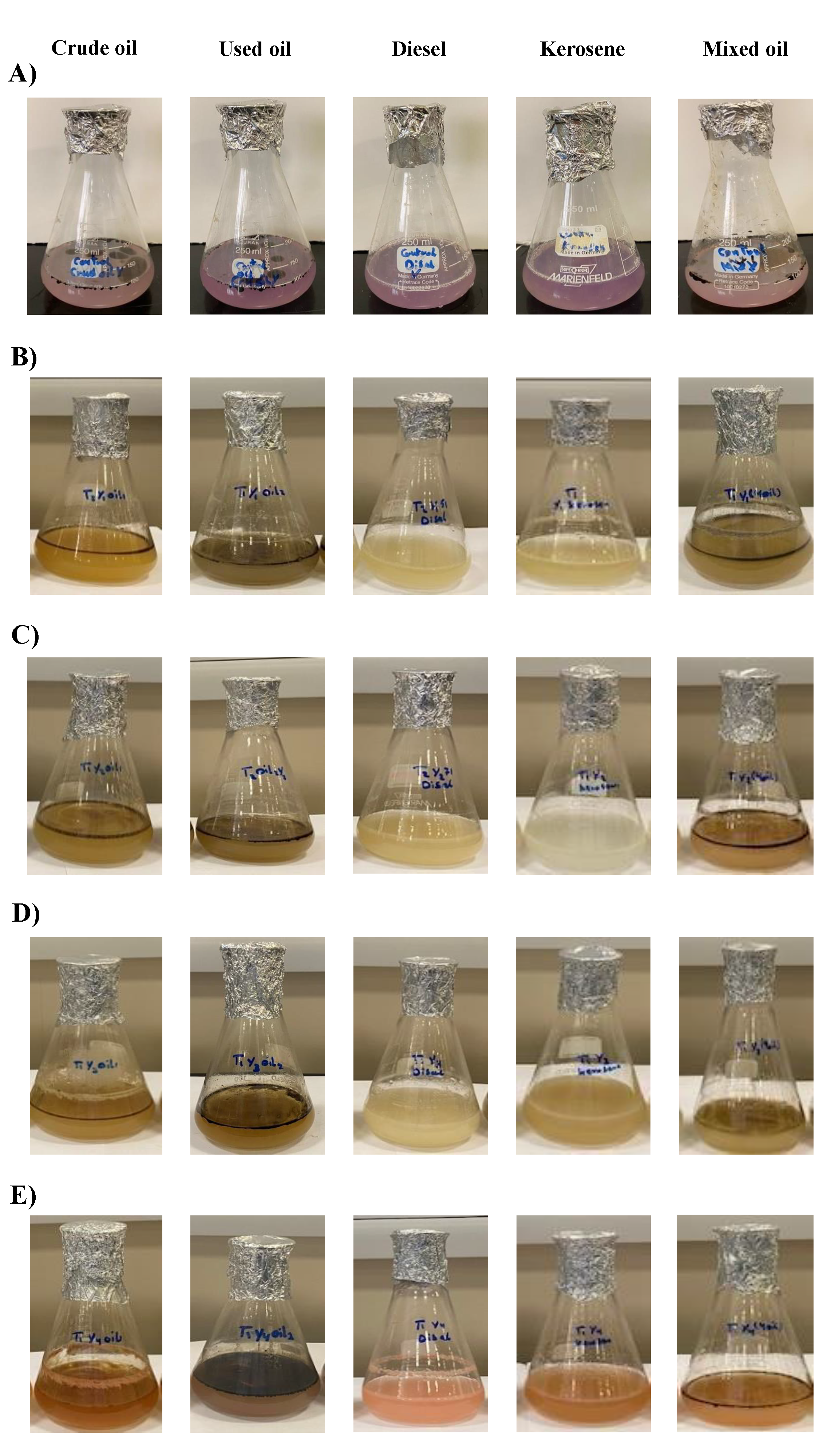

Supplement: Supplementary file 3 — Supplementary Information 3. [file 41598_2022_14836_MOESM3_ESM.tiff]

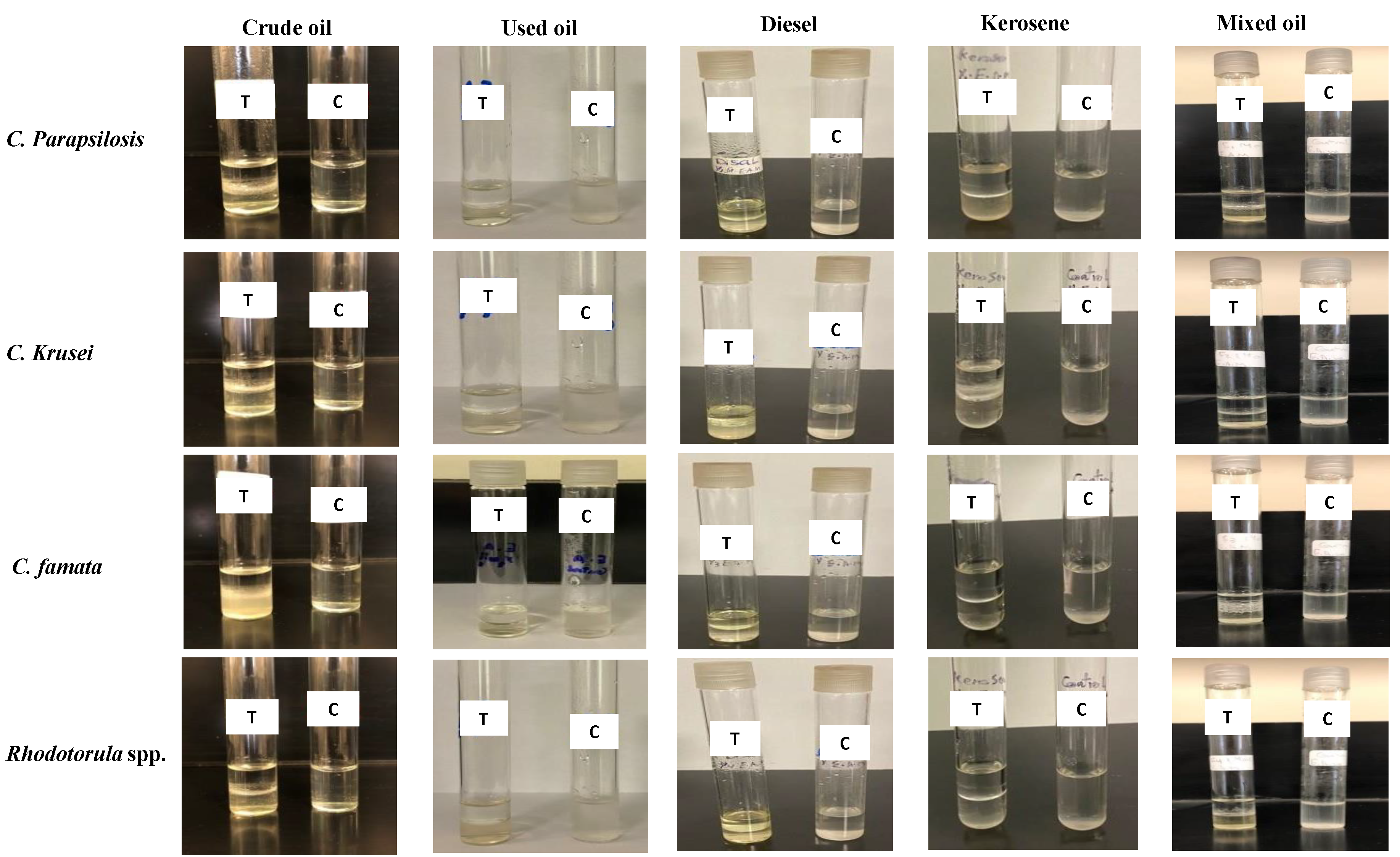

Supplement: Supplementary file 4 — Supplementary Information 4. [file 41598_2022_14836_MOESM4_ESM.tiff]
