# Supplementary material for: The crude oil biodegradation activity of Candida strains isolated from oil-reservoirs soils in Saudi Arabia
Source: Sci Rep. 2022 Jun 23;12:10708. doi: 10.1038/s41598-022-14836-0 (PMC9226172; doi:10.1038/s41598-022-14836-0)
Supplement: Supplementary file 5 — Supplementary Information 5. [file 41598_2022_14836_MOESM5_ESM.docx]

**Supplementary Figure 1. Growth of different fungi in liquid media.** MSM contained 1% various oil sources and was incubated in a shaker at 25°C for 30 days. (A) *C. parapsilosis,* (B) *C. krusei,*(C) *C. famata,*and (D) *Rhodotorula* spp.

**Supplementary Figure 2.** **The oil biodegrading assay of tested isolates.**The isolates were grown on liquid media (MSM) containing 1% various oil sources; 0.1% (v/v) of Tween 80, 0.6 mg/mL of redox indicator (DCPIP), and incubated in a shaker at 25°C for 15 days. (A) control, (B) *C. parapsilosis*, (C) *C. krusei,* (D) *C. famata*, and (E) *Rhodotorula*spp.

**Supplementary Figure 3. Emulsification activity assay.**The emulsification activity measured the ability of the different isolated fungal strains to emulsify different hydrocarbons. Equal volumes of CFSs and different hydrocarbons were mixed and incubated for 24h at 25°C, which resulted in the separation of two layers in the treated tubes (Left), as compared to the control (Right).
